# Unilateral Giant Varicocele Mimicking Inguinal Hernia Resulting from Portosystemic Shunt without Evidence of Portal Hypertension: An Unusual Case Report

**DOI:** 10.1155/2013/709835

**Published:** 2013-02-28

**Authors:** Muhammed Zahir, Hassan R. Al Muttairi, Surjya Prasad Upadhyay, Piyush N. Mallick

**Affiliations:** Al Jahra Hospital, Ministry of Health, State of Kuwait, P.O. Box 40206, 01753 Safat, Kuwait

## Abstract

Isolated giant varicocele has been reported with portal hypertension that results in abnormal communication between portal venous system and testicular vein venous system resulting in retrograde backflow of blood into the testicular venous system which leads to varicosity of the pampiniform plexuses. 65-year-old male with no past medical or surgical history presented to us with soft inguinoscrotal swelling that disappears on lying down mimicking inguinal hernia. Clinical examination revealed soft inguionoscrotal swelling that disappears on pressure. Ultrasonography revealed varicosity of pampiniform plexus, and CT angiography to trace the extent of the varicosity revealed abnormal communication of right testicular vein with superior mesenteric vein. There was no evidence of any portal hypertension; the cause of the portosystemic shunt remains obscure, and it might be a salvage pathway for increasing portal pressure. The case is noteworthy for its rare presentation and abnormal communication with portal venous system in the absence of evidence of portal hypertension.

## 1. Introduction 

Simple inguinal hernia presents as lump in the groin that goes away with lying down or with minimal pressure. Most cases may be painless or cause mild to moderate discomfort that increases with activity [1]. Varicocele result from abnormal dilatation of pampiniform venous plexus of the scrotum that drained testicular venous blood and ascent through the inguinal canal to drain into inferior vena cava (on right side) or renal vein (left side). Retrograde flow of blood into these venous channels can occur in various conditions such as absence of venous valve or incompetent valve or abnormal venous communication or pressure due to obstruction/compression upstream of the venous draining resulting in tortuosity and dilatation of vein [2]. Abnormal dilatation may also mimic hydrocele [3], or inguinal hernia. Simple varicocele is easily diagnosed by clinical examination; ultrasonography, Doppler imaging, and venography can be used to diagnose latent or complicated varicocele [4]. Most of the varicocele in clinical practice are on left side. We encountered a case of giant varicocele presented as gross inguinoscrotal swelling on right side mimicking inguinal hernia which used to disappear on lying down. 

## 2. Case Report

**Figure 1:**
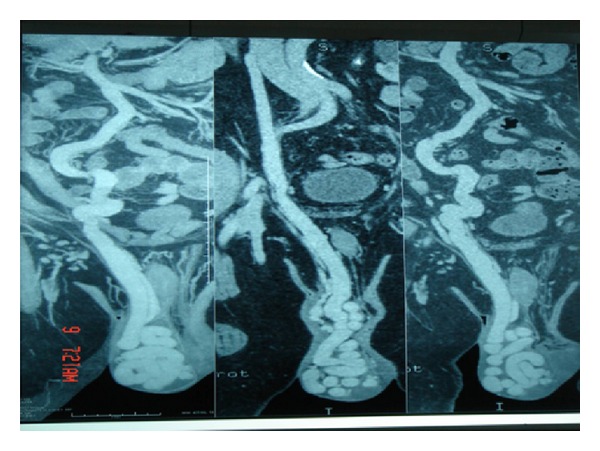
CT angiography showing right testicular vein draining into inferior vena cava and abnormal communication with superior mesenteric vein.

**Figure 2:**
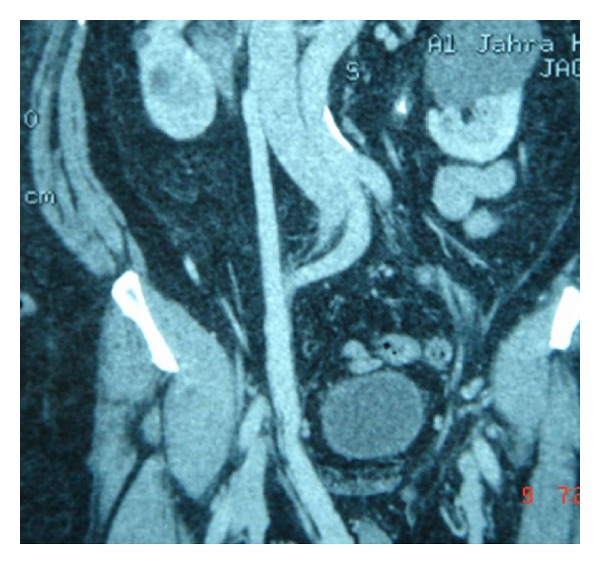
Abnormal communication of superior mesenteric vein and rt testicular vein.

A 65-year-old, Indian male, presented to urology outpatient with history of reducible swelling of right scrotum and groin associated with dull aching and dragging sensation that started couple of years ago. He was nonalcoholic, married, and having three children. There was no medical or surgical history of illness or trauma in the past. Examination of the genitalia revealed large painless compressible soft swelling of right inguinoscrotal region which completely disappears while lying down. Left side scrotum was absolutely normal. Ultrasound and Doppler scrotum were done as initial evaluation which reported a huge right sided varicocele. CT angiography was done which showed two communications in the venous drainage system, that is, one right testicular vein channel draining into the inferior vena cava (normal) and another large abnormal connection with superior mesenteric vein (Figures 1 and 2). There was no history or clinical finding or radiological evidence suggesting any portal hypertension or any condition that might lead to portal hypertension. 

We consulted vascular surgeon for possible embolisation of the venous communication but patient refused such intervention. He is on regular followup in our clinic with no further deterioration of his clinical condition.

## 3. Discussion

Isolated large varicocele may be secondary to multiple intraabdominal pathology, such as malignancy causing venous compression, portal hypertension or portal vein thrombosis, abnormal venous communications, or heart failure [5]. The pampiniform venous plexus ascents and coalesces along the spermatic cord to form gonadal vein (testicular vein) which drained into inferior vena cava on right side and into renal vein on left side. Multiple venous collateral communications may arise, usually between the gonadal vein origin and deep inguinal ring. These are parallel or perpendicular to the gonadal vein and may include retroperitoneal, perirenal, and lumbar veins [6, 7]. Development of varicocel in portal hypertension can be explained as portosystemic shunt that provides salvage pathways for increasing portal pressure [2, 3, 8].

In our case there was abnormal communication of right testicular vein and superior mesenteric vein in our patient without any clinical signs and symptoms or any evidence of portal hypertension; the cause of the abnormal communication still remains obscure, and it might be a salvage pathway for raising portal pressure.

Our case was unique in presentation also; there was no typical finding of “bag of worm” in scrotum which is diagnostic in varicocele. The inguinoscrotal swelling was soft and used to disappear with pressure or lying down mimicking inguinal hernia. 

Treatment of such pathological condition has not been defined; some surgeon advised to avoid surgery in such situation [8] for fear of massive unanticipated blood loss or opening of other portosystemic shunt. The abnormal communication might have served as salvage pathway for increasing portal pressure because of which there might not be signs and symptoms of portal hypertension. We planned for embolization of the communicating vessel but patient refused the intervention.

## 4. Conclusion

Isolated giant varicocele resulting from abnormal communication of right testicular vein and superior mesenteric vein and mimicking inguinal hernia is extremely rare condition. Surgical intervention in such situation might result in unanticipated blood loss or rupture of another portosystemic shunt. The definitive management of such condition is still to be defined.
